# Routine Pharmacological Venous Thromboembolism (VTE) Prophylaxis in Lower-Limb Injuries: A Three-Year Retrospective Cross-Sectional Study and Service Evaluation

**DOI:** 10.7759/cureus.97088

**Published:** 2025-11-17

**Authors:** Mahmoud Mersal, Jija Thomas, Mohamed Elmarakby, Mohamed Alsonbaty, Osama Embaby, Abdelrahman Embabi, Nouman Zubair, Donald Buchanan

**Affiliations:** 1 Trauma and Orthopaedics, University Hospitals Birmingham (UHB) Foundation Trust, Birmingham, GBR; 2 Trauma and Orthopaedics, Sandwell and West Birmingham Hospitals National Health Service (NHS) Trust, Birmingham, GBR; 3 Clinical Emergency Medicine, Royal Surrey National Health Service (NHS) Foundation Trust, London, GBR

**Keywords:** apixaban, bleeding, deep vein thrombosis (dvt), enoxaparin, lower limb immobilisation, pulmonary embolism (pe), venous thromboembolism

## Abstract

Background

Lower-limb injury and immobilisation substantially increase the risk of venous thromboembolism (VTE). This study assessed outcomes following the introduction of routine pharmacological VTE prophylaxis for patients with lower-limb injuries at a UK teaching hospital.

Aim

The aim of the study is to evaluate three years of routine VTE prophylaxis in lower-limb injuries, focusing on inpatient thromboembolic events, bleeding outcomes, and prescribing patterns.

Methods

This retrospective study reviewed consecutive patients with lower-limb injuries managed in a single UK teaching trust following the introduction of routine pharmacological VTE prophylaxis. Data were extracted from the hospital’s Prescribing Information and Communication System (PICS) covering the period 2022-2024. Data represented all lower-limb injury admissions over a three-year period rather than longitudinal follow-up of individual patients. The primary outcomes were symptomatic VTE events (deep vein thrombosis (DVT) or pulmonary embolism (PE)) during hospitalisation, recorded bleeding episodes during hospitalisation, and prescribing patterns of prophylaxis agents.

Results

A total of 4,011 patients were included (mean age 53.4 ± 21.1 years, and 54.6% were women). Enoxaparin was the predominant prophylactic agent (86.5%), followed by apixaban (5.9%). Bleeding-related entries were identified in 36.0% of patients. No DVT cases were identified, and one PE was recorded during the three-year study period.

Conclusion

Routine VTE prophylaxis, predominantly low-molecular-weight heparin (LMWH)-based with limited direct oral anticoagulant (DOAC) use, appears effective in substantially reducing thromboembolic events in lower-limb injuries, though inpatient bleeding entries were common but ungraded.

## Introduction

Background

Lower-limb fractures and immobilisation are well-established risk factors for venous thromboembolism (VTE), encompassing both deep vein thrombosis (DVT) and pulmonary embolism (PE) [1,2]. Immobilisation contributes to venous stasis, endothelial dysfunction, and hypercoagulability, which are the key elements of Virchow’s triad, thereby predisposing patients to thrombosis [2]. The risk is further amplified in the presence of fracture, soft-tissue trauma, or prolonged non-weight-bearing status.

Historically, VTE prophylaxis was selective, reserved for patients deemed high-risk based on factors such as age, obesity, or comorbidities [3,4]. However, modern guidance has shifted towards a universal approach, recommending routine pharmacological prophylaxis for all lower-limb injury patients with reduced mobility [4]. This reflects growing evidence that broad prophylactic coverage effectively reduces thromboembolic events without significant bleeding complications.

Over the past decade, multiple large studies and meta-analyses have reinforced the benefit of prophylaxis. A systematic review showed that low-molecular-weight heparin (LMWH) significantly reduced the risk of any VTE and symptomatic DVT compared with no prophylaxis [5]. A 2022 network meta-analysis of 14 studies (8,198 patients) demonstrated that rivaroxaban, fondaparinux, and LMWH each reduced VTE risk substantially, with rivaroxaban ranked highest for efficacy [6].

Observational data from Tran et al. and Kayali et al. confirmed that untreated lower-limb immobilisation carries a 1%-2% symptomatic VTE risk, whilst rivaroxaban and LMWH prophylaxis markedly reduce this rate [2,7]. Public and professional awareness campaigns, such as those led by Thrombosis UK, have also supported broader adoption of routine prophylaxis in immobilised patients [8]. In surgical cohorts, such as hip-fracture patients, VTE risk remains substantial despite mobility recovery, further validating the importance of consistent prophylaxis [9].

Risk stratification and rationale

Attempts to predict VTE risk through scoring systems have shown variable accuracy. The TIP and TRiP(cast) models identified age and injury type as the most reproducible predictors, but their overall discriminatory power remains limited [10,11]. Consequently, many experts advocate universal pharmacological prophylaxis for immobilised lower-limb injuries. 

Despite strong evidence, real-world evaluations of outcomes following the introduction of routine prophylaxis remain scarce. This study aimed to evaluate outcomes following the introduction of routine pharmacological VTE prophylaxis for lower-limb injuries, describe prescribing patterns, and assess the frequency of thromboembolic and bleeding events over a three-year period.

## Materials and methods

Study design and setting

This retrospective study, registered under code: CARMS-20693, was conducted at University Hospitals Birmingham (UHB) NHS Foundation Trust, a large UK university teaching hospital. The dataset included all adult patients admitted between January 1, 2022, and December 31, 2024, with lower-limb fractures or injuries coded as the primary diagnosis on their admitting episode.

Data were obtained from two hospital information systems: the Oceano database, which provided coded diagnostic and demographic details for identifying lower-limb injury admissions, and the Prescribing Information and Communication System (PICS), which supplied prescribing data and automated ‘Yes/No‘ diagnostic flags based on keywords such as ‘PE‘ and ‘DVT‘. Both the Oceano and PICS systems record events documented during hospitalisation only; post-discharge or outpatient events are not routinely captured or linked. Because these flags captured both confirmed and negated mentions (e.g., ‘no evidence of PE‘), they were considered unreliable for definitive outcome verification and used only for initial screening. In accordance with the National Data Opt-Out policy, 237 records were excluded prior to analysis.

Continuous variables were summarised as mean ± SD and categorical variables as frequencies (percentages). In this study, ‘incidence’ refers to newly identified VTE events occurring during hospitalisation within the defined three-year study period; confirmed VTE was reported as both raw counts and per-1,000-patient rates. Statistical analysis and data cleaning were performed.

Inclusion and exclusion criteria

All patients aged 16 years or older who were admitted with lower-limb injuries requiring immobilisation or inpatient management were included in the study. Patients whose primary reason for admission was a non-orthopaedic condition were excluded from the analysis.

Outcomes

The primary outcome was the occurrence of confirmed symptomatic VTE, DVT or PE, prior to discharge from the hospital. Secondary outcomes included the documentation of bleeding events, identified by any flag for bleeding, haematuria, haematemesis, or haemoptysis, and the distribution and frequency of pharmacological prophylaxis agents prescribed.

## Results

A total of 4,011 patients were included in the analysis. The cohort comprised 54.6% women (n = 2,192) and 45.4% men (n = 1,819), with a mean age of 53.4 ± 21.1 years (median 52, interquartile range 35.5-71.0) (Table 1).

**Table 1:** Demographic and clinical characteristics of patients admitted with lower-limb injuries IQR: interquartile range

Variable	n (%) or mean ± SD
Total patients	4,011
Female	2,192 (54.6%)
Male	1,819 (45.4%)
Mean age (years)	53.4 ± 21.1
Median age (IQR)	52 (35.5–71.0)

All patients received pharmacological thromboprophylaxis according to the institutional protocol. Enoxaparin was the predominant agent, prescribed in 3,471 cases (86.5%), followed by apixaban in 235 (5.9%), whilst 305 (7.6%) records were unspecified or missing drug data (Table 2).

**Table 2:** Distribution of pharmacological VTE prophylaxis agents among patients with lower-limb injuries The table summarises the prescribing patterns of pharmacological thromboprophylaxis in the study cohort VTE: venous thromboembolism

Agent	n (%)
Enoxaparin	3,471 (86.5%)
Apixaban	235 (5.9%)
Unspecified/missing record	305 (7.6%)

Thromboembolic and bleeding events

During the three-year period, no cases of DVT were confirmed, and one case of PE (0.025%) was identified. Bleeding-related entries were recorded in 1,445 patients (36.0%). These were automatically generated symptom flags from clinical documentation and were not clinically validated or severity graded. Therefore, they are likely to include false positives and minor, self-limiting events rather than confirmed haemorrhagic complications.

## Discussion

Following the introduction of routine pharmacological prophylaxis for patients with lower-limb injuries, our centre observed no cases of inpatient DVT and only one case of inpatient PE among more than 4,000 admissions during the three-year study period. This policy was associated with an extremely low observed rate of symptomatic VTE events and an acceptable observed safety profile, given the absence of clinically confirmed major haemorrhagic complications.

Our findings are consistent with extensive evidence supporting the efficacy of pharmacological thromboprophylaxis in preventing VTE among patients with lower-limb injuries. The systematic review by Horner et al. demonstrated an approximate 50% reduction in VTE risk with LMWH compared with no prophylaxis [5]. Similarly, the network meta-analysis by Douillet et al. confirmed significant risk reduction with rivaroxaban, fondaparinux, and LMWH, with rivaroxaban showing the greatest efficacy [6]. These findings align with the landmark randomised trial by Kakkar et al., which demonstrated that extended-duration prophylaxis markedly reduced post-immobilisation VTE events in trauma and orthopaedic patients [12]. Although bleeding-related entries were frequent (36%), these reflect automated keyword-generated PICS flags rather than confirmed haemorrhagic events. Previous large-scale randomized controlled trials (RCTs) have reported major bleeding rates between 0.2% and 1.5% [6,12], suggesting that the present figure likely represents an overestimation due to data-capture artefact rather than true clinical harm.

Real-world data from Tran et al. and Kayali et al. further validated the effectiveness of LMWH and direct oral anticoagulants (DOACs) in trauma and immobilisation settings [2,7]. In addition, the TiLLI review by Horner et al. summarised the clinical and cost-effectiveness evidence supporting routine thromboprophylaxis in patients with temporary lower-limb immobilisation, reinforcing the rationale for universal prophylaxis policies in such populations [13]. The Cochrane review by Testroote et al. also noted that even early, selective prophylaxis protocols conferred measurable benefit [3].

Risk-prediction tools such as the TIP [10] and TRiP(cast) [11] scores have yet to demonstrate sufficient discriminatory accuracy to replace universal prophylaxis strategies. Moreover, evidence from hip-fracture cohorts continues to highlight a persistently elevated VTE risk even after mobility is restored [9]. Collectively, these findings support the use of broad, routine pharmacological prophylaxis, particularly LMWH-based regimens, as an effective and safe approach to minimising VTE risk in patients with lower-limb injuries.

Strengths and limitations

This study’s strengths include its large consecutive cohort of more than 4,000 patients, providing strong statistical reliability and real-world relevance. It reflects routine clinical practice within a high-volume UK trauma centre, enhancing generalisability to similar institutions. Consistent adherence to the prophylaxis protocol across the three-year study period further supports the validity of the findings.

However, several limitations should be acknowledged. The analysis relied on automatically generated PICS flags, which may have resulted in under- or overestimation of certain outcomes due to negated or ambiguous documentation. Importantly, only inpatient VTE and bleeding events were captured, with no post-discharge follow-up, which likely underestimates the true event rates. The retrospective, single-centre design and absence of a control group also limit causal inference and generalisability beyond this institutional setting.

Clinical implications

Routine LMWH-based prophylaxis in lower-limb injuries appears to be both safe and highly effective in real-world practice. Institutions should prioritise accurate outcome documentation, particularly regarding bleeding, and evaluate the potential role of DOACs as alternative prophylactic agents for selected patients.

Future directions

Future research should include multicentre prospective comparisons of LMWH versus DOACs, evaluation of optimal prophylaxis duration, and robust cost-effectiveness analyses contrasting universal and risk-stratified approaches.

## Conclusions

Implementation of routine pharmacological VTE prophylaxis for lower-limb injuries with limited mobility was associated with no DVTs and only one PE over a three-year period. These findings support current guideline recommendations for routine prophylaxis in immobilised lower-limb injury patients. Whilst the results demonstrate a strong association between universal pharmacological prophylaxis and low in-hospital VTE rates, further prospective research is warranted to confirm causality and evaluate post-discharge outcomes.
